# Progress Toward Poliomyelitis Eradication — Nigeria, January 2013–September 2014

**Published:** 2014-11-21

**Authors:** Andrew Etsano, Rajni Gunnala, Faisal Shuaib, Eunice Damisa, Pascal Mkanda, Richard Banda, Charles Korir, Ogu Enemaku, Melissa Corkum, Samuel Usman, Lora B. Davis, Gatei wa Nganda, Cara C. Burns, Frank Mahoney, John F. Vertefeuille

**Affiliations:** 1 National Primary Health Care Development Agency, Federal Republic of Nigeria; 2 Global Immunization Division, Center for Global Health, CDC; 3 Federal Ministry of Health, Federal Republic of Nigeria; 4 World Health Organization, Nigeria Office; 5 United Nations Children’s Fund, Nigeria Office; 6 CORE Group Partners Project Nigeria; 7 Division of Viral Diseases, National Center for Immunization and Respiratory Diseases, CDC

In 1988, the World Health Assembly resolved to interrupt wild poliovirus (WPV) transmission worldwide (1). By 2013, only three countries remained that had never interrupted WPV transmission: Afghanistan, Nigeria, and Pakistan. Since 2003, northern Nigeria has been a reservoir for WPV reintroduction into 26 previously polio-free countries. In May 2014, the World Health Organization declared the international spread of polio a Public Health Emergency of International Concern. Nigeria’s main strategic goal is to interrupt WPV type 1 (WPV1) transmission by the end of 2014 (2), which is also a main objective of the Global Polio Eradication Initiative’s Polio Eradication and Endgame Strategic Plan for 2013–2018 (3). This report updates previous reports (4–6) and describes polio eradication activities and progress in Nigeria during January 2013–September 30, 2014. Only six WPV cases had been reported in 2014 through September 30 compared with 49 reported cases during the same period in 2013. The quality of supplemental immunization activities (SIAs)* improved during this period; the proportion of local government areas (LGAs) within 11 high-risk states† with estimated oral poliovirus vaccine (OPV) campaign coverage at or above the 90% threshold increased from 36% to 67%. However, the number of reported circulating vaccine-derived poliovirus type 2 (cVDPV2) cases increased from four in 2013 to 21 to date in 2014, and surveillance gaps are suggested by genomic sequence analysis and continued detection of WPV1 by environmental surveillance. Interrupting all poliovirus circulation in Nigeria is achievable with continued attention to stopping cVDPV2 transmission, improving the quality of acute flaccid paralysis (AFP) surveillance, increasing vaccination coverage by strengthened routine immunization services, continuing support from all levels of government, and undertaking special initiatives to provide vaccination to children in conflict-affected areas in northeastern Nigeria.

## Vaccination Activities

Routine immunization for infants and children in Nigeria includes vaccination with trivalent OPV (tOPV) at birth and at ages 6, 10, and 14 weeks. The 2013 Nigeria Demographic and Health Survey§ reported national coverage with 3 doses of trivalent oral polio vaccine (OPV3)¶ of children aged 12–23 months at 38.2% (7). OPV3 coverage estimates among the northern 11 high-risk states ranged from 2.6% for Sokoto to 43.7% for Kaduna.

During January 2013–September 2014, 24 SIAs were implemented. Three national SIAs used tOPV (the last one occurring in August 2014), two national SIAs used bivalent OPV (bOPV), and 19 subnational SIAs used bOPV, mostly in high-risk states. A major focus for SIA implementation has been in two transmission “zones”: the “Kano zone,” which includes LGAs (equivalent to districts) in the south of Kano as well as LGAs in northeastern Kaduna and northwestern Bauchi, and the “Borno/Yobe zone,” which includes Borno and Yobe (8). In Kano, intensified SIA plans include statewide microplanning validated by walk-through and, consistent with national policy, responding to any new WPV as though it were an outbreak. During June–October, three outbreak response campaigns were implemented in response to each of the three most recent WPV1 cases detected in Kano, supplementing subnational SIAs.

In Borno and Yobe, the innovations being implemented to address challenges caused by insecurity include the use of “permanent health teams” comprised of women who deliver OPV to households within their own communities, transit-point vaccination, vaccination in camps for internally displaced persons, and “short-interval” SIAs that take advantage of transient access to normally inaccessible areas. In June and August 2014, inactivated polio vaccine was included along with tOPV in SIAs conducted in 27 LGAs of Borno and Yobe, vaccinating an estimated 1.7 million children aged 14 weeks to 5 years (8). Plans are under way to include inactivated polio vaccine along with OPV in two SIAs during November–December 2014 for the remaining 12 LGAs in Borno/Yobe and 13 high-risk LGAs in the Kano transmission zone. A national strategy to increase SIA implementation quality also has included multi-intervention health camps** to build community confidence in government health programs.

**FIGURE 1:**
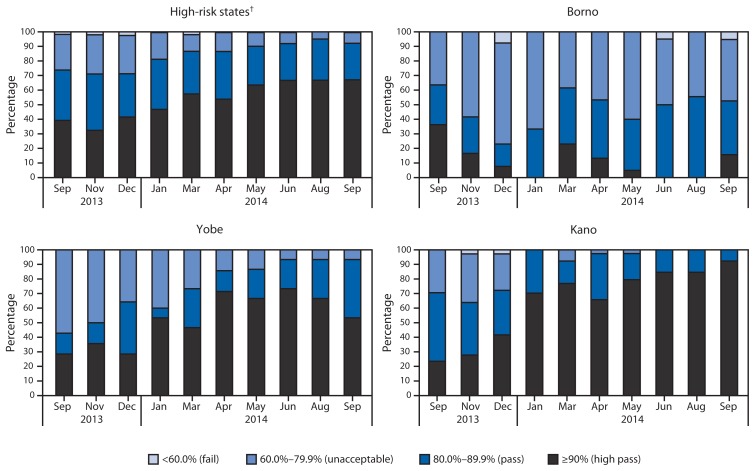
Percentage of local government areas with indicated quality category from lot quality assurance sampling (LQAS*) surveys assessing supplementary immunization activities, by state and month — northern Nigeria, September 2013–September 2014 * LQAS surveys are used to assess the quality of polio supplemental immunization activities (SIAs) in local government areas, using a four-category pass/fail scheme based on the proportion of children with a finger mark indicating they had recieved oral poliovirus vaccine during the SIA. ^†^ Bauchi, Borno, Jigawa, Kaduna, Kano, Katsina, Kebbi, Niger, Sokoto, Yobe, and Zamfara.

The quality of SIAs is assessed using lot quality assurance sampling (LQAS)†† surveys to estimate whether OPV coverage thresholds have been met. During February 2013–September 2014, the number of LGAs conducting LQAS in the 11 high-risk states increased from 168 to 218; the proportion of LGAs at the ≥90% OPV coverage threshold increased from 36% to 67%, the proportion of LGAs at the 80%–89% threshold decreased from 29% to 25%, and the proportion of LGAs below the 80% threshold decreased from 36% to 7%. (Figure 1).

## Poliovirus Surveillance

### AFP surveillance

Polio surveillance relies on AFP case detection and confirmation of polio by laboratory viral isolation. The two primary performance indicators for AFP surveillance are a nonpolio AFP (NPAFP) rate of ≥2 cases per 100,000 children aged <15 years per year and collection of adequate stool specimens in ≥80% of AFP cases (4). The annualized NPAFP rate for 2014 was 14.4 per 100,000, and 98.8% of AFP cases had adequate stool specimen collection. This is higher than the 2013 NPAFP rate of 12.1 cases per 100,000, and 96.9% of AFP cases with adequate stool collection. All of the 11 high-risk states exceeded both indicator standards in 2013 and have continued to do so in 2014. The proportion of LGAs within these states that met both standards increased from 91.8% in 2013 to 99.3% in 2014.

### Environmental surveillance

AFP surveillance is supplemented by environmental surveillance, with samples taken from effluent sewage sites every 4–5 weeks for poliovirus testing. By September 2014, environmental surveillance was conducted in 27 sites: Borno (four sites), Kaduna (three sites), Kano (three sites), Lagos (five sites), Sokoto (four sites), the Federal Capital Territory (two sites), Kebbi (three sites), and Katsina (three sites). During January–September 2014, WPV1 was identified in one sewage sample collected in May in Kaduna. In 2013, WPV1 was detected in four sewage samples (one from Kano in February, two from Sokoto in March and April, and one from Borno in October). WPV type 3 (WPV3) was last detected in a sewage sample from a site in Lagos in November 2012; cVDPV2 has been detected repeatedly in sewage samples from Sokoto and Borno since mid-2013, and in Kano and Kaduna since April 2014.

What is already known on this topic?

Nigeria is one of three countries worldwide where wild poliovirus (WPV) transmission has never been interrupted. Historically, poor public health infrastructure and poor-quality immunization activities have been considered responsible for failure of interruption, and strategies to combat such issues have been put in place in recent years, resulting in considerable programmatic improvements.

**What is added by this report?**

For the period January–September, WPV case incidence decreased dramatically from 49 in 2013 to six in 2014. However, transmission of circulating vaccine-derived poliovirus continues. Transmission of type 1 WPV is localized to two “transmission zones”: Kano and Borno/Yobe, where supplemental immunization activities are being intensified. Quality of supplemental immunization activities has improved, as have acute flaccid paralysis surveillance indicators, but data suggest some surveillance gaps might still exist.

**What are the implications for public health practice?**

Nigeria has the potential to interrupt polio transmission in 2014, thus removing itself as a reservoir of WPV. National program innovations and strategies to improve polio vaccine coverage for underserved and hard-to-reach communities have resulted in measurable successes. The final steps toward polio eradication in Nigeria will require continuation of these efforts. If eradication becomes a reality, lesson learned and resources used towards this effort can be redirected towards addressing other national public health issues.

## Wild Poliovirus Incidence

### WPV and cVDPV incidence

**FIGURE 2:**
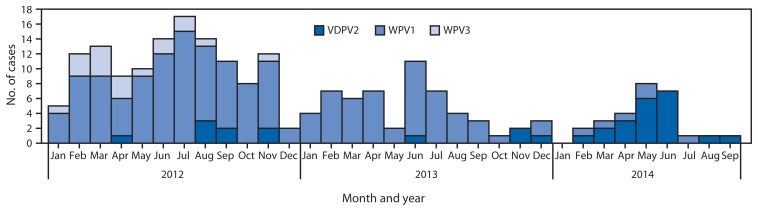
Number of reported cases of wild poliovirus type 1 (WPV1), wild poliovirus type 3 (WPV3), and vaccine-derived poliovirus type 2 (VDPV2), by month — Nigeria, January 2012–September 2014

**FIGURE 3:**
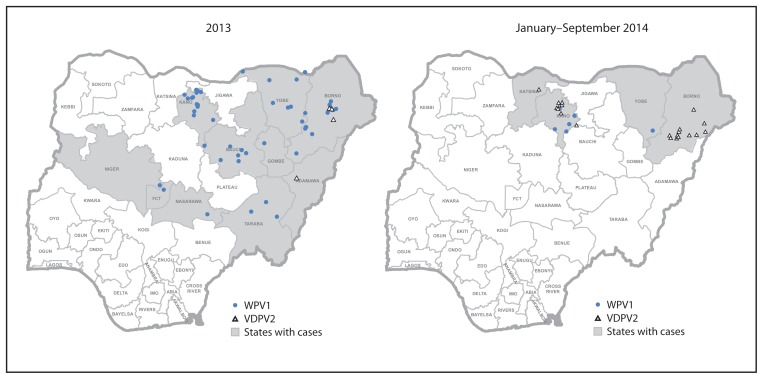
Distribution of reported cases of wild poliovirus type 1 (WPV1) and vaccine-derived poliovirus type 2 (VDPV2)*, by state — Nigeria, 2013 and January–September 2014 * Each dot represents one case placed at random within a local government area boundary. No cases of wild poliovirus type 3 were reported.

As of September 2014, six WPV cases had been reported nationally, compared with 49 WPV cases for the same period in 2013. Reported cases decreased from 122 in 2012 to 53 in 2013. No WPV3 cases have been reported since November 2012 (Figures 2 and 3). WPV1 cases in 2014 have been limited to five cases in the “Kano transmission zone” (onset of most recent case on July 24, 2014) and one case in the “Borno/Yobe transmission zone” (onset of most recent case on April 19, 2014). Incidence of cVDPV2 cases varied from 10 in 2012 to four in 2013, to 21 in 2014 to date (12 in Borno, eight in Kano, one in Katsina).

### Genomic sequence analysis

WPV genetic diversity in Nigeria declined during January 2013–September 2014. Eight genetic clusters of poliovirus were detected in 2012; of these, four were detected in 2013. Two genetic clusters detected in 2013 have been detected so far in 2014. Genomic sequence analysis can also be used to indicate AFP surveillance gaps not otherwise shown by surveillance performance indicators if poliovirus isolates have a nucleotide difference of ≥1.5% in the coding region of the major capsid protein, VP1, from the closest matching sequences of previously identified isolates (4). The number of WPV1 isolates with a nucleotide difference of ≥1.5% was 10 (of 103 isolates sequenced) in 2012, 10 (of 53) in 2013, and two (of six) to date in 2014.

### Discussion

WPV incidence declined substantially in Nigeria during 2013–2014 coincident with a concerted effort of the national polio eradication program in coordination with global partners. In particular, during the high transmission season of June–September, reported cases declined 96% from 24 cases during 2013 to one case in 2014 (8). No WPV3 cases or environmental isolates have been identified since November 2012, indicating possible interruption of WPV3. SIA quality as assessed by LQAS surveys of OPV coverage has improved nationally, and multiple strategies are being implemented to target hard-to-reach communities and decrease vaccine refusals. Intensified implementation of SIAs is being focused on the “Kano” and “Borno/Yobe” transmission zones, with the intention of interrupting the last remaining chains of WPV1 transmission by the end of 2014.

Despite meeting AFP surveillance performance indicators at national and subnational levels thus far in 2014, genomic sequencing analysis and continued detection of WPV1 in environmental surveillance strongly suggest that surveillance gaps at subnational levels remain. Improved standardization of surveillance activities at state and LGA levels is warranted.

With the main focus on prioritizing interruption of WPV transmission and the predominant use of bOPV during the majority of SIAs conducted during January 2013–September 2014, cVDPV2 incidence has increased. Two SIAs planned for the remainder of 2014 will use tOPV, and inactivated polio vaccine will be added in highest-risk LGAs in transmission zones to boost population immunity to levels needed to interrupt cVDPV2 transmission.

Some longstanding challenges to achieving polio eradication in Nigeria remain, and new challenges have emerged. Although the proportion of children nationally who received all vaccines based on national age-specific recommendations increased from 13% in 2003 to 25% in 2013 (7) and OPV3 coverage has improved nationally, routine vaccination coverage has remained well below targeted coverage levels. The 11 high-risk states in particular have historically low coverage and will likely benefit from planned routine immunization intensification strategies, including a “hard-to-reach” project. This project aims to increase coverage in vulnerable and underserved areas by delivering polio vaccine along with other interventions aimed at preventing and treating childhood pneumonia, diarrhea, malaria, and other vaccine-preventable disease.

The strong support from all levels of government for polio eradication will need to be sustained and intensified, particularly as insecurity continues to restrict access to children during SIAs in areas of Borno, Yobe, and northern Adamawa. In addition, the emergence of widespread Ebola viral disease throughout West Africa has put a strain on health care infrastructure and personnel across the region. The recent Ebola outbreak in Nigeria was successfully interrupted in part because the polio eradication response infrastructure was used; in particular, members of the Nigeria Polio Emergency Operations Center were deployed to coordinate the multi-agency Ebola response (9). Continuing active management and addressing ongoing challenges can create the potential for a WPV-free African continent.

## Footnotes

* Mass campaigns conducted for a few days during which 1 dose of oral poliovirus vaccine is administered to all children aged <5 years, regardless of vaccination history. Campaigns can be conducted nationally or subnationally.

† Bauchi, Borno, Jigawa, Kaduna, Kano, Katsina, Kebbi, Niger, Sokoto, Yobe, and Zamfara.

¶ DTP3 coverage used as surrogate for routine immunization coverage since reported OPV can include doses given during SIAs.

§ These routine immunization coverage estimates differ from administrative data presented in previous *MMWR* updates because these data come from the 2013 Nigeria Demographic and Health Survey. This survey was conducted using a nationally representative sample of 40,320 households and was designed to produce reliable estimates for key indicators at the national level as well as for urban and rural areas, each of the country’s six geographic zones, and each of the 36 states and the Federal Capital Territory. More information on survey methodology available at http://dhsprogram.com/publications/publication-FR293-DHS-Final-Reports.cfm.

** Fixed-point vaccination centers providing a variety of primary health services during SIAs.

†† A clustered LQAS methodology is used to assess SIA quality by sampling the target population of children at the LGA level and documenting finger markings indicative of OPV receipt. A sample is drawn from six wards (geopolitical subunits) within the LGA, with 10 children in a single settlement selected at random from each sampled ward. This yields a total sample of 60 children per LGA. LGAs are classified into one of four classifications based on the number of unmarked children found: 0–3 dark green (high pass); 4–8 light green (pass); 9–19 yellow (unacceptable); >19 red (fail). A detailed description of the methodology is available at http://www.polioeradication.org/Portals/0/Document/Research/OPVDelivery/LQAS.pdf.
